# An *APC* Mutation in a Large Chinese Kindred With Familial Adenomatous Polyposis Was Identified Using Both Next Generation Sequencing and Simple STR Marker Haplotypes

**DOI:** 10.3389/fgene.2020.00191

**Published:** 2020-03-04

**Authors:** Qitao Zhan, Liya Wang, Xiangrong Xu, Yan Sun, Lejun Li, Xuchen Qi, Feng Chen, Xiaoming Wei, Michael L. Raff, Ping Yu, Fan Jin

**Affiliations:** ^1^Key Laboratory of Reproductive Genetics (Ministry of Education), Department of Reproductive Endocrinology, Women’s Hospital, Zhejiang University School of Medicine, Hangzhou, China; ^2^Department of Obstetrics and Gynecology, Beijing Tongren Hospital, Capital Medical University, Beijing, China; ^3^Department of Neurosurgery, Sir Run Run Shaw Hospital, Zhejiang University School of Medicine, Hangzhou, China; ^4^BGI-Shenzhen, Shenzhen, China; ^5^Genomics Institute, MultiCare Health System, Tacoma, WA, United States; ^6^Department of Cell Biology and Medical Genetics, Zhejiang University School of Medicine, Hangzhou, China

**Keywords:** familial adenomatous polyposis, *APC* gene, STR marker, next generation sequencing, mutation

## Abstract

**Background:**

Familial adenomatous polyposis (FAP) is an autosomal dominant disorder characterized primarily by the development of numerous adenomatous polyps in the colon and a high risk for colorectal cancer. FAP is caused by germline mutations of the adenomatous polyposis coli (*APC*) gene. The proband in this family was a 39-year-old female patient with the pathologic diagnosis of adenomatous polyps, and then a five-generation kindred with FAP was characterized in the following years. This article identified an *APC* mutation, and demonstrated the practical use of *APC*-linked STR markers, which could be used to reduce misdiagnosis of prenatal diagnosis or preimplantation genetic diagnosis resulted from contamination or allele drop-out.

**Methods:**

Next-generation sequencing (NGS) was used to identify the possible *APC* mutations in an affected individual from a family with autosomal dominant colon cancer. Targeted sequencing then used to identify additional related individuals with the mutation. Three short tandem repeat (STR) loci, D5S299, D5S134, and D5S346, were used for PCR-based microsatellite analysis of the *APC* gene in the extended family.

**Results:**

We identified an *APC*: p.W553X mutation. The STR haplotype at the *APC* locus, A1B4C1, was shared by all clinically affected individuals with the *APC*: p.W553X mutation. In addition, the *APC*: p.D1822V variant was observed in 40% affected individuals and in two unaffected individuals.

**Conclusion:**

We described a protein truncation mutation, *APC*: p.W553X; demonstrated the value of *APC*-linked STR markers (D5S299, D5S134, and D5S346) haplotypes; and suggested the potential role of these haplotypes in detecting loss of heterozygosity of the *APC* gene.

## Introduction

Familial adenomatous polyposis (FAP) is an autosomal dominant disorder characterized primarily by the development of numerous adenomatous polyps in the colon and a high risk for colorectal cancer (Koskenvuo et al., 2016). Without a proper diagnosis to ensure heightened colon surveillance and timely surgical intervention, affected individuals are likely to develop colorectal adenocarcinoma. FAP is a clinically heterogeneous disorder, including variation in the age of onset, presence of extracolonic lesions, and the number, distribution, and histology of colorectal adenomas. Even within a single affected pedigree, there is also significant clinical variability.

Familial adenomatous polyposis is caused by germline mutations of the adenomatous polyposis coli (*APC*) gene, located on chromosome 5q21-22 (van der Luijt et al., 1995; Grover et al., 2012). The coding region of *APC* encompasses 8532 bp that was distributed among 15 exons. *APC* gene mutations have been identified in 60–80% of all FAP families and remain unknown in 20–30% (Friedl et al., 2001). Large deletions that evade detection by conventional DNA screening methods, mutations located in introns, regulatory regions or splice-acceptor site, and mutations in other unidentified genes are possible explanations for this low *APC* gene mutation detection rate (Stekrova et al., 2007; Zhang et al., 2016, 2017; Wang et al., 2017). The use of next-generation sequencing (NGS) has revolutionized the way of mapping a mutation and identifying its molecular identity, leading to massive improvements in our understanding on the role of nucleic acids functions (Doitsidou et al., 2016; Del Vecchio et al., 2017). Targeted sequencing of candidate cancer genes and whole-exome and whole-genome sequencing, coupled with encouraging clinical results based on the use of targeted therapeutics and biomarker-guided clinical trials, are fueling further technological advancements of NGS technology (Khotskaya et al., 2017). Nevertheless, the use of informative polymorphic markers linked to the *APC* gene may still be valuable in the diagnoses of FAP family members.

Short-tandem repeats (STR) are highly polymorphic two-to-five base pair CA repeat units interspersed throughout the genome. The information provided by polymorphic markers can be markedly increased by constructing haplotypes-defined patterns of alleles at closely linked polymorphic loci. STR markers D5S318 (Wijnen et al., 1991; Gonzalez et al., 2005), D5S299 (van Leeuwen et al., 1991; Wijnen et al., 1991), D5S82 (Breukel et al., 1991; Wijnen et al., 1991), D5S134 and D5S346 are located within 1–10 cM of the *APC* gene (Wijnen et al., 1991; Flintoff et al., 2001). Of these, D5S134 is within 1 cM of the 5′ end of the gene, and D5S346 is tightly linked within 30–70 kb of the 3′ end of the gene (Wong et al., 1996; Barth et al., 1997). If a particular haplotype of these markers can be linked to the FAP phenotype in a pedigree, genetic diagnosis of pre-symptomatic individuals is possible without requiring an identified *APC* gene mutation. Furthermore, if a particular haplotype can be linked to a pathogenic *APC* gene mutation, misdiagnosis of prenatal diagnosis or preimplantation genetic diagnosis resulted from contamination or allele drop-out could be avoided.

In this large kindred, we firstly confirmed the disease through the pathology results. Then we analyzed the whole data of a five-generation kindred with 100 control individuals to find and verify disease related mutations. We used NGS to identify possible *APC* mutations, and employed *APC*-linked STR marker analysis to complete the molecular investigation of the five-generation family with the *APC* mutations.

## Method

### Patients

**FIGURE 1:**
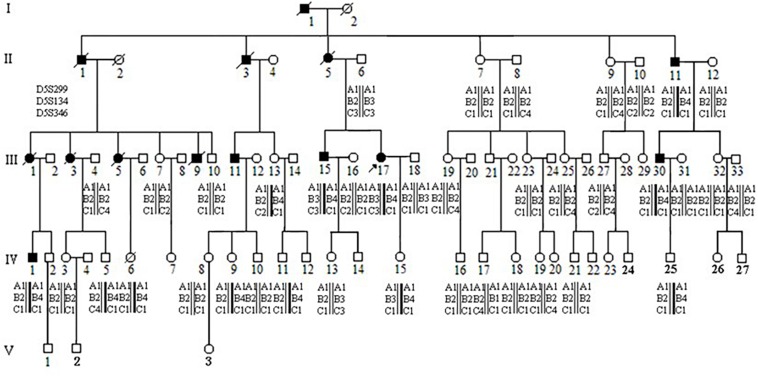
Pedigree and haplotype analysis with STR markers D5S299, D5S134, D5S346. Individual III-17 (arrow) is the proband. By PCR and electrophoresis, D5S299 alleles are designated A1-4; D5S134 alleles, B1-4; and D5S346 alleles, C1-4. Haplotype A1B4C1 (bold) segregates with the disease phenotype.

The proband (III-17), a 39-year-old female patient with the pathologic diagnosis of adenomatous polyps by local hospital, went to the First Affiliated Hospital of Medical School of Zhejiang University for further treatment. Colonoscopy revealed that the surface of her colon and rectum segment was covered with polyps. There were about 55 polyps per 5 cm in the ileocecal region and about 25 polyps per 5 cm in the relatively sparse area. Then the patient underwent total colorectal resection and gastroduodenostomy with ileal pouch. Microscopic examination showed that the largest polyp, at a distance of 29 cm from the upper incision, showed tubular adenoma change with grade II-III epithelial dysplasia. The rest of the polyps showed tubular adenoma change with grade I-II epithelial dysplasia. Pathological diagnosis was FAP. After the recovery, this patient came to Women’s Hospital, School of Medicine, Zhejiang University, for genetic counseling of preimplantation genetic diagnosis, and became the first to be characterized with *APC*: p.W553X mutation and *APC*: p.D1822V variant in this family. Figure 1 shows the pedigree of a five-generation family with FAP. The diagnosis of FAP in affected individuals was based on colonoscopy or histological verification after colectomy. Affected individuals are shaded black (II-11, III-11, III-15, III-17, III-30, and IV-1). The clinical and molecular characterization of individual family members is shown in Table 1. In all, 30 descendants of the first generation and nine biologically unrelated spouses were studied at the molecular level.

**TABLE 1:** Clinical characteristics of individual family members.

**Individual**	**Gender**	**Age (years)**	**Clinical phenotype**	**Genotype Codon 553**	**Genotype Codon 1822**
II-6	Male	69	–	WT	WT
II-7	Female	60	–	WT	WT
II-8	Male	59	–	WT	WT
II-9	Female	53	–	WT	WT
II-10	Male	55	–	WT	WT
II-11	Male	56	+	W553X	WT
II-12	Female	51	–	WT	WT
III-4	Male	50	–	WT	WT
III-7	Female	41	–	WT	WT
III-10	Male	35	–	WT	WT
III-13	Female	41	–	W553X	WT
III-15	Male	46	+	W553X	D1822V
III-16	Female	39	–	WT	D1822V
III-17	Female	39	+	W553X	D1822V
III-18	Male	40	–	WT	WT
III-19	Female	40	–	WT	WT
III-23	Female	34		WT	WT
III-25	Female	30		WT	WT
III-27	Male	30		WT	WT
III-30	Male	30	+	W553X	WT
III-31	Female	26		WT	WT
III-32	Female	26	–	WT	WT
III-33	Male	28		WT	WT
IV-1	Male	28	+	W553X	WT
IV-3	Female	26		WT	WT
IV-5	Male	20		W553X	WT
IV-6	Female	21		W553X	WT
IV-8	Female	19		WT	WT
IV-9	Female	13		W553X	WT
IV-10	Male	10		WT	WT
IV-11	Male	20		W553X	WT
IV-13	Female	15		WT	D1822V
IV-15	Female	15		W553X	WT
IV-16	Male	15		WT	WT
IV-17	Male	15		WT	WT
IV-18	Female	13		WT	WT
IV-19	Female	9		WT	WT
IV-21	Male	13		WT	WT
IV-25	Male	5		W553X	WT

### Morphological Analysis

Individual III-17 underwent a preventive total colectomy in 1998, and the 85 cm resected colon and rectum was studied. Individual III-15 underwent a total colectomy in 1997, and the 65 cm resected colon was studied. The tissues were fixed in 4% paraformaldehyde and embedded in wax. Sections 5 μm thick were stained with hematoxylin and eosin, and observed using an Olympus BX51 microscope.

### Detection of *APC* Gene Mutation

DNA sample was obtained from affected individual III-17 (Figure 1), a 39 year old female with prior colectomy, and sequenced using microarray-based NGS. A custom Sequence Capture 2.1 M Human Array from Roche NimbleGen (Madison, United States) was designed to capture a total of 1.5 Mb of DNA containing 3093 exons (including 100 bp of flanking DNA) of 222 genes associated with common genetic diseases, including *APC*. The preparation of libraries was consistent with published standard operating protocols (Gnirke et al., 2009). In each pooled batch, 10 to 33 samples were sequenced simultaneously on Illumina HiSeq2000 Analyzers (Illumina, San Diego, United States) for 90 cycles. Image analysis, error estimation, and base calling were performed using Illumina Pipeline software (version 1.3.4). Raw sequence reads were screened following established filtering criteria. Clean reads with a length of 90 bp were aligned to the reference human genome from the NCBI database (GRCh37) using the Burrows Wheeler Aligner (BWA) Multi-Vision software package with output files in BAM format. The BAM data were used for reads coverage in the target region and sequencing depth computation, SNP and INDEL calling, and CNV detection. The *APC* mutation detected in the proband was not found in 100 control individuals. Direct DNA sequencing was used to determine the FAP mutation status of additional family members.

### Sanger Sequencing

Sanger sequencing was then carried out to confirm the identified mutations by exome sequencing. Specific primers for amplifying exon 13 of the *APC* gene (NM_001127511) were designed using the Primer 3.0 (primer sequencing: 13-Forward CAG CCT CCC AAA GTG ATA GG, and 13-Reverse ATG GCT AAA AGA AGG CAG CA). DNA was amplified in a 20 μl reaction volume: 10 μl of PCR mix (2× HS Taq PCR Mix, Takara, Japan), 0.2 μM of each primer, 100 ng genomic DNA and 7.2 ul of DNase/RNase free water. The PCR cycling profile was as follow: initial denaturation at 94°C for 5 min, followed by 30 cycles at 94°C for 30 s, 56°C for 30 s, and 72°C for 30 s, and a final extension at 72°C for 10 min. The PCR products were checked on 2% agarose (w/v) gel. Sequence analysis was run on an ABI 3730XL DNA Analyzer (Applied Biosystems, United States).

### Polymorphism in Codon 1822 Confirmed by Restriction Enzyme *Bpi*I

*APC*: p.D1882V was assayed in all family member and 37 control individuals by restriction enzyme *Bpi*I (5′-GAAGAC(N)2↓-3′, 3′-CTTCTG(N)6↓-5′).

### Linkage Analysis

Three short tandem repeat (STR) loci, D5S299 (GDB:180445), D5S134 (GDB:196678), and D5S346 (GDB:181171), were used for PCR-based microsatellite analysis of the *APC* gene. After amplification, PCR products were separated by 8% native polyacrylamide gel electrophoresis at 800 v for 4–5 h and DNA fragments detected with silver staining. Based on a decreasing migration rate of PCR product, the amplification products of D5S346 were named A1, A2, A3, and A4; D5S134 products were named B1, B2, B3, and B4; D5S299 were named C1, C2, C3, and C4.

## Results

### Morphological Analysis

#### III-17

**FIGURE 2:**
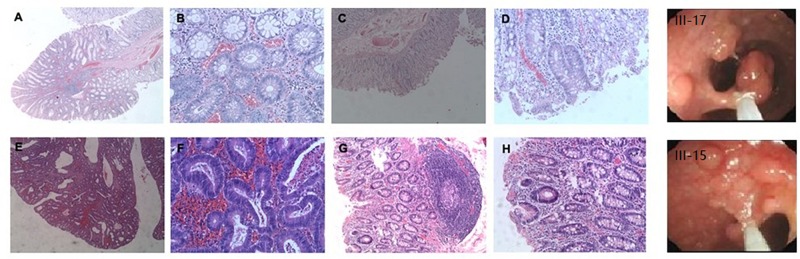
Adenomatous polyps in colorectum of individual III-17 and III-15. **(A–D)**, Polyps in III-17. **(E–H)** Polyps in III-15. **(A,B,E,F)** Typical tubular adenomas or microadenomas. **(C,D,G,H)** Characteristic single dysplastic crypts.

There were approximately 15 small polyps with a diameter range of 0.1–0.6 cm. Histologically, these polyps were typical tubular adenomas or microadenomas with low grade intra-epithelial neoplasia (Figures 2A,B). The characteristic single dysplastic crypts (unicryptal adenomas), the initiation of adenomas, were occasionally found in the colon mucosa (Figures 2C,D).

#### III-15

There were more than 500 polyps with diameters ranging from 0.1 to 2.0 cm in the colon. Histologically, these polyps appeared as tubular adenomas or microadenomas (Figure 2E^∗^50). Most adenomas showed low grade intra-epithelial neoplasia except for the largest one which was high grade (Figure 2F^∗^200). The characteristic single dysplastic crypt (unicryptal adenomas) was occasionally found in the colonic mucosa (Figures 2G^∗^100, H^∗^200).

### Mutation Detection

**FIGURE 3:**
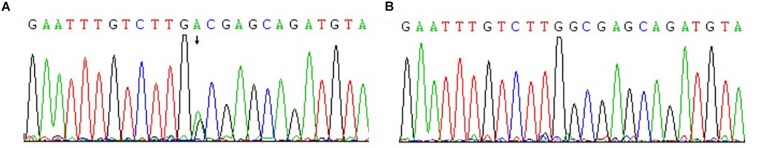
Confirmation of novel G-to-A mutation (arrow) by DNA sequencing. **(A)** The affected proband (III-17); **(B)** An unaffected family member.

Microarray-based NGS of the proband (individual III-17 in Figure 1) revealed a novel heterozygous mutation, TGG > TGA (Figure 3). This nonsense mutation substitutes a stop codon for a tryptophan codon (W553X). Direct sequencing identified the same mutation in 11 additional individuals across three generations of the extended family (II-11, III-13, III-15, III-30, IV-1, IV-5, IV-6, IV-9, IV-11, IV-15, and IV-25 in Figure 1), five of whom (II-11, III-15, III-17, III-30, and IV-1) manifest clinical findings of familial adenomatous polyposis, others could not finished the examinations because of the young age or disagreement of their parents.

The proband’s STR haplotype at the *APC* locus, A1B4C1, was shared by all clinically affected individuals as well as all individuals, affected and unaffected, with the *APC*: p.W553X mutation (Figure 1).

Interestingly, the *APC*: p.D1822V variant was observed linked to the A1B3C3 haplotype but was not seen in individual II-6, who passed on this haplotype to his two children. These two children had also inherited clinical findings of FAP from their affected mother (II-5) as well as (by indirect evidence) the A1B3C3 haplotype and the *APC*: p.W553X mutation. This result implied that the *APC*: p.D1822V variant had arisen anew in individual II-6 linked to the A1B3C3 haplotype and was present presumably as a gonadal mosaic, which was consistent with previous studies (Iwaizumi et al., 2015; Kahyo et al., 2017).

## Discussion

Familial adenomatous polyposis is a hereditary colorectal cancer syndrome that arises from germline mutations in the *APC* tumor suppressor gene (Lamlum et al., 1999). This study describes a novel c.1659G > A mutation that creates a stop codon at a tryptophan codon–W553X–in the APC protein. The resulting truncated protein product lacks all axin- and β-catenin-binding sites and is therefore presumed defective in the regulation of β-catenin phosphorylation and ubiquitination (Fearnhead et al., 2001). Such a truncated protein might instead function as an activator for Wnt signaling which provides a strong selective advantage by affecting cell proliferation, migration, apoptosis and possibly differentiation of intestinal stem cells (Zhang et al., 2012).

We identified this mutation in 12 individuals among 30 tested in a multi-generational family with familial adenomatous polyposis. Of these twelve, five are clinically affected by the disorder. Among the seven asymptomatic mutation carriers, one (III-13) is 41 years old, while the others are all under 21 years of age. 95% of individuals who have inherited an *APC* mutation will manifest clinical findings by age 35 years, while only 50% will have manifested findings by age 16 years (Petersen et al., 1991; Aihara et al., 2014). Thus, because of the asymptomatic 41 year old, this family displays incomplete penetrance (Evans et al., 1993). Halling et al. reported two families with truncated mutations at codons 1982–1983 and 1982 with desmoid tumors, which are observed in 10% of FAP families (Halling et al., 1999) but with incomplete penetrance for the development of intestinal polyps 0.16 Even when there is complete penetrance, considerable variation can be seen among FAP patients, both in the spectrum of extracolonic features and in the extent of intestinal polyposis (Fearon, 1997; Koskenvuo et al., 2016). Dinucleotide repeat microsatellite markers D5S299, D5S134, and D5S346, are located immediately adjacent to the *APC* gene (Koorey et al., 1992; Gray et al., 2011). In this study, all five clinically affected individuals in our FAP extended family shared the same haplotype for these markers: A1B4C1. All were confirmed to carry the *APC*: p.W553X mutation. Seven additional family members (III-13, IV-5, IV-6, IV-9, IV-11, IV-15, and IV-25) were found to have the same A1B4C1 haplotype. The presence of the *APC*: p.W553X mutation in these individuals confirmed the accuracy of the haplotyping. These seven individuals are at high risk for developing colorectal cancer and should receive appropriate surveillance, including annual colonoscopy 0.1 This includes the individual who is asymptomatic at 41 years of age (III-13), as non-penetrance cannot be assumed with certainly, and the two adolescent individuals (IV-9 and IV-15). This does not yet include the 5 year old individual (IV-25) as screening does not begin until 10–15 years of age. These STR markers can also be used to detect loss of heterozygosity of the *APC* locus in tumor tissue from colon cancer as well as from other tumors where loss of the *APC* gene is implicated, such as gastric cancer, non-small cell lung cancer, renal cell cancer, endometrial cancer, and squamous cell carcinoma.

We observed that the frequency of the *APC*: p.D1822V variant allele in this family was not higher than in the healthy control population (10% vs. 18.9%), consistent with its reported lack of clinical significance (Ruiz-Ponte et al., 2001; Tranah et al., 2005). This missense variant changes a hydrophilic asparate to a hydrophobic valine residue between the fourth and fifth of the seven 20-amino-acid repeats involved in the binding and down-regulation of β-catenin (Polakis, 1997). Efficient β-catenin down-regulation requires a minimum of three of the seven binding repeats (coinciding with the 3′ limit of the mutation cluster region), suggesting that the *APC*: p.D1822V polymorphism does not have an appreciable effect on β-catenin degradation (Tranah et al., 2005). This variant is reported in the exome variant server with a frequency of 21%.

Obviously, STR linkages were prominent in this large kindred, but there were no extended evidences for whether these STRs were really useful to search for detecting new *APC* mutations in other families. They would be useful in LOH (loss of heterozygosity) research, but this would be so when we investigate somatic change in tumors arising in the patients of W553X carriers. Besides, in our study, we were hoping for new discoveries in this FAP family; however, most of these genes were not targeted to FAP and indeed there were no new related findings.

In summary, microarray-based NGS and direct sequencing identified a novel mutation, *APC*: p.W553X, in a large Chinese kindred with familial adenomatous polyposis. In addition, the value of *APC*-linked STR marker (D5S299, D5S134, and D5S346) haplotypes was demonstrated in tracking inheritance of *APC* alleles, and their use in uncovering loss of heterozygosity of the *APC* gene suggested for future studies.

## Data Availability Statement

The raw data supporting the conclusions of this article will be made available by the authors, without undue reservation, to any qualified researcher.

## Ethics Statement

The studies involving human participants were reviewed and approved by the Key Laboratory of Reproductive Genetics of Women’s Hospital, Zhejiang University School of Medicine. Written informed consent to participate in this study was provided by the participants or their legal guardian/next of kin.

## Author Contributions

QZ, LW, and FJ designed the study and performed the major genetic experiments. XX, YS, and PY performed the molecular biological experiments and the analysis of pathological examination. FC was in charge of specimen collection and finished the patients’ follow-ups. XW carried out the next generation sequencing. QZ and XQ wrote the manuscript. YS, LL, and MR revised the manuscript.

## Conflict of Interest

The authors declare that the research was conducted in the absence of any commercial or financial relationships that could be construed as a potential conflict of interest. The reviewer SB declared a shared affiliation, though no other collaboration, with one of the authors PY to the handling Editor.
